# Supplementary material for: MetaRibo-Seq measures translation in microbiomes
Source: Nat Commun. 2020 Jun 29;11:3268. doi: 10.1038/s41467-020-17081-z (PMC7324362; doi:10.1038/s41467-020-17081-z)
Supplement: Supplementary file 10 — Supplementary Data 7 [file 41467_2020_17081_MOESM10_ESM.zip › File2/Confidence_High_Taxonomy/276144_out.krona.html]

Javascript must be enabled to view this page.

members
magnitude
magnitudeUnassigned
count
unassigned
taxon
rank

276144\_out

50

50
superkingdom
2

976
phylum
50

200643
class
50

50
171549
order

815
family
50

816
genus
50

6
1263054
species

SRS011405\_contig\_number\_2657SRS015217\_contig\_number\_29805SRS017701\_contig\_number\_16765SRS053649\_contig\_number\_6742SRS078419\_contig\_number\_36128SRS101376\_contig\_number\_4298

39

SRS013800\_contig\_number\_22861SRS016753\_contig\_number\_1496SRS017521\_contig\_number\_48451SRS017821\_contig\_number\_9339SRS018133\_contig\_number\_3184SRS018817\_contig\_number\_9836SRS019496\_contig\_number\_3893SRS019787\_contig\_number\_1355SRS022609\_contig\_number\_16285SRS042284\_contig\_number\_725SRS045195\_contig\_number\_contig-100\_822.63319SRS045645\_contig\_number\_8026SRS046068\_contig\_number\_994SRS046712\_contig\_number\_332SRS049446\_contig\_number\_461SRS049959\_contig\_number\_35820SRS052697\_contig\_number\_25974SRS062654\_contig\_number\_17435SRS063040\_contig\_number\_22407SRS065504\_contig\_number\_11153SRS077127\_contig\_number\_11484SRS077231\_contig\_number\_23299SRS097958\_contig\_number\_21800SRS098073\_contig\_number\_3740SRS098827\_contig\_number\_8363SRS098881\_contig\_number\_8911SRS1041039\_contig\_number\_22406SRS1041143\_contig\_number\_946SRS1041144\_contig\_number\_7428SRS104311\_contig\_number\_24675SRS1055038\_contig\_number\_4103SRS1055095\_contig\_number\_2053SRS140492\_contig\_number\_contig-100\_1994.180527SRS143372\_contig\_number\_2800SRS147272\_contig\_number\_34SRS148874\_contig\_number\_5100SRS893253\_contig\_number\_822SRS893256\_contig\_number\_1160SRS893300\_contig\_number\_11451
species
818

1
371601
species

SRS049402\_contig\_number\_13899

3

SRS024388\_contig\_number\_12481SRS074670\_contig\_number\_4423SRS076804\_contig\_number\_19386
species
46506

1

SRS104327\_contig\_number\_1565
species
47678
